# Supplementary material for: Confirmatory Study on Costs and Time Loss from Pre-Anesthetic Consultations for Canceled Surgeries: A Retrospective Analysis at Hannover Medical School, Germany
Source: J Clin Med. 2025 Sep 13;14(18):6454. doi: 10.3390/jcm14186454 (PMC12470992; doi:10.3390/jcm14186454)
Supplement: Supplementary file 1 [file jcm-14-06454-s001.zip › jcm-3844995-supplementary.pdf]

Confirmational study about the Costs and Time Loss from Pre-Anesthesia Consultations for Canceled Surgeries: A Retrospective Study at Hannover Medical School in Germany

Steffen B Wiegand, Anna S. Heinemann, Dennis Harries, David Bürger, Lisa Thiehoff, Anna Fischbach

Table S1. Remuneration table according to German TV-Ärzte as of September 2023.

Remuneration group	Pay group 1	Pay group 2	Pay group 3	Pay group 4	Pay group 5	Pay group 6
Resident physician	EUR 5,104.24 1st-year	EUR 5,393.56 2nd-year	EUR 5,600.21 3rd-year	EUR 5,958.42 4th-year	EUR 6,385.47 5th-year	EUR 6,552.04 6th-year
Specialist	EUR 6,736.78 1st-year	EUR 7,301.63 4th-year	EUR 7,797.59 7th-year	EUR 8,076.29 9th-year	EUR 8,228.22 11th-year	EUR 8,438.20 13th-year
Attending physician	EUR 8,438.20 1st-year	EUR 8,934.16 4th-year	EUR 9,643.64 7th-year	/	/	/
Chief physician	EUR 9,926.10 1st-year	EUR 10,635.56 4th-year	EUR 11,200.40 7th-year	/	/	/

Table S2. Duration and cost of PAC consultation in the NoANE group depending on specialty.

Specialty	All patients n = 86	Duration of PAC [min/case]	Cost of PAC [EUR/case] ± SD
Neurosurgery	2 (2.3 %)	73.5 ± 54.5	39.14 ± 30.10
Gynecology	20 (23.3 %)	34.4 ± 15.8	23.91 ± 13.69
General Surgery	2 (2.3 %)	41.0 ± 5.7	22.96 ± 3.17
Dentistry/ Maxillofacial Surgery	5 (5.8 %)	35.4 ± 25.2	20.75 ± 15.12
Otorhinolaryngology	11 (12.8 %)	34.4 ± 15.9	19.99 ± 9.82
Neurology	1 (1.2 %)	28.0 ± 0.0	18.76 ± 0.00
Internal Medicine	7 (8.1 %)	31.0 ± 24.9	18.11 ± 13.54
Urology	4 (4.7 %)	31.0 ± 5.5	17.89 ± 3.23
Trauma Surgery	4 (4.7 %)	25.5 ± 8.5	16.78 ± 7.85
Thoracic Surgery	2 (2.3 %)	24.5 ± 7.8	15.22 ± 3.51
Ophthalmology	17 (19.8 %)	22.6 ± 9.4	13.62 ± 5.76
Pediatrics	7 (8.1 %)	20.1 ± 4.5	12.80 ± 2.63
Cardiac Surgery	4 (4.7 %)	18.5 ± 2.6	12.67 ± 2.89
Dermatology	0 (0 %)	0.0 ± 0.0	0.00 ± 0.00
Vascular Surgery	0 (0 %)	0.0 ± 0.0	0.00 ± 0.00

Nuclear Medicine/ Radiology	0 (0 %)	0.0 ± 0.0	0.00 ± 0.00
Plastic Surgery	0 (0 %)	0.0 ± 0.0	0.00 ± 0.00
Psychiatry	0 (0 %)	0.0 ± 0.0	0.00 ± 0.00
**Overall**	**86 (100 %)**	**30.1 ± 17.3**	**18.75 ± 11.17**
***p*-Value**		**0.012**	**0.005**

Results are expressed as mean ± SD or No. (%). PAC = pre-anesthesia consultation.
